# Subcellular Min Oscillations as a Single-Cell Reporter of the Action of Polycations, Protamine, and Gentamicin on *Escherichia coli*

**DOI:** 10.1371/journal.pone.0007285

**Published:** 2009-09-30

**Authors:** Benjamin P. B. Downing, Andrew D. Rutenberg, Ahmed Touhami, Manfred Jericho

**Affiliations:** Department of Physics and Atmospheric Science, Dalhousie University, Halifax, Nova Scotia, Canada; The University of Manchester, United Kingdom

## Abstract

**Background:**

In *Escherichia coli*, MinD-GFP fusion proteins show rapid pole to pole oscillations. The objective was to investigate the effects of extracellular cations on the subcellular oscillation of cytoplasmic MinD within *Escherichia coli*.

**Methodology/Principal Findings:**

We exposed bacteria to the extracellular cations Ca^++^, Mg^++^, the cationic antimicrobial peptide (CAP) protamine, and the cationic aminoglycoside gentamicin. We found rapid and substantial increases in the average MinD oscillation periods in the presence of any of these polyvalent cations. For Ca^++^ and Mg^++^ the increases in period were transient, even with a constant extracellular concentration, while increases in period for protamine or gentamicin were apparently irreversible. We also found striking interdependence in the action of the small cations with protamine or gentamicin, distorted oscillations under the action of intermediate levels of gentamicin and Ca^++^, and reversible freezing of the Min oscillation at high cationic concentrations.

**Conclusions/Significance:**

Intracellular Min oscillations provide a fast single-cell reporter of bacterial response to extracellular polycations, which can be explained by the penetration of polycations into cells.

## Introduction

Within *E. coli*, Min proteins undergo subcellular oscillations [1] that target division to midcell. The basic biochemistry of the Min oscillation is understood. Cytoplasmic MinD:ATP binds to the plasma membrane and recruits MinE to it. MinE stimulates the intrinsic MinD:ATPase, and the subsequent hydrolysis releases MinD and MinE back into the cytoplasm. MinD:ATP then undergoes nucleotide exchange in the cytoplasm.

Min oscillations at room temperature have a period of about 40 s [2], and a spatial wavelength in filamentous cells of about 8 microns [3]. The oscillation period depends on mutations of MinE [4], on the proportion of MinD to MinE [3], and on the ambient temperature [5]. The variation of oscillation period with temperature has been attributed to variations of the MinE-stimulated MinD-ATPase activity [5].

In *E. coli*, Mg^++^ is needed for ATP association with MinD [6], [7], for MinD ATPase activity [6], for membrane association [8]–[10], and for MinD polymerization *in vitro* [8], [9]. Ca^++^ is necessary for ATPase activity of the MinD-homologue AtMinD1 in plastids [11], but is not required in *E. coli*. MinD is associated with the inner leaflet of the cytoplasmic membrane. However, since intracellular ion concentrations are often influenced by extracellular concentrations, one might expect that extracellular multivalent cations affect Min oscillations *in vivo*. In this paper we have begun to explore the response of the Min oscillation to extracellular multivalent cations.

Ca^++^ is implicated in a number of bacterial functions, including chemotaxis and the cell-cycle [12], [13]. Recombinant aequorin protein has offered an elegant way to measure free intracellular Ca^++^ concentration ([Ca^++^]_i_) [14], [15], but measurements on individual cells has not yet been achieved. Typical [Ca^++^]_i_ is at least a few hundred nM [14] and depends transiently on the extracellular Ca^++^ concentrations [15]. Homeostasis of the cytoplasmic Ca^++^ concentrations is observed: with a constant cytoplasmic steady-state concentration eventually recovered after extracellular concentrations are changed [15]. Survivability of *E. coli* in a wide range of external Ca^++^ concentrations ranging from µM to tens of mM has been demonstrated.

Mg^++^ is a necessary cofactor for many enzymatic reactions and is actively regulated by bacteria [16], [17]. Total cellular Mg^++^ is approximately 100 mM while free intracellular [Mg^++^]_i_ is approximately 1 mM [18], a thousandfold higher than typical [Ca^++^]_i_. There are not yet recombinant reporters of [Mg^++^]_i_, analogous to aequorin, though there are synthetic fluorescent probes (see, e.g., [19]). For bacterial growth tens of µM extracellular Mg^++^ is sufficient, and growth continues with external concentrations of hundreds of mM.

The multifaceted action of antimicrobial agents on cells, inhibiting growth and leading towards cell death, has been investigated extensively. Despite this, basic questions such as how cytoplasmically acting antimicrobial agents penetrate into the cytoplasm are still being debated (see e.g. [20]). One reason for this is that there have been no intracellular reporters for small amounts of antimicrobial agents *in vivo*. Many antimicrobial agents have lytic properties, especially at higher concentrations. However, at lower concentrations many also appear to translocate into the cytoplasm without cell death and have significant intracellular effect. We investigate the effect, without lysis, of two polycationic antimicrobial agents on Min oscillations: the aminoglycoside gentamicin [21] and the antimicrobial peptide protamine [22], [23]. Commercial preparations of gentamicin [24] contain mixes of three molecular varieties with *Mr*s (relative molar masses) of 478, 450, and 464. Gentamicin is positively charged at physiological pH and carries a charge of 3.5+ at a pH of 7.4 [24]. The minimum inhibitory concentration (MIC, where net growth is zero) of gentamicin for *E. coli* is 1 µg/ml [25]. Protamine, with 20 arginine residues and a molecular weight of 4112Da, has a minimum bactericidal concentration (MBC, where net growth is negative) of 153 µM and a MIC of 75 µM in *E. coli* strain 25922 [26]. The effect of protamine on food borne bacteria including *E. coli* was investigated by Potter *et al.* [26].

In this paper we report a slowing of the cytoplasmic Min oscillations in response to all of the tested extracellular polycations. We propose that Min oscillations can be used as a fast single-cell reporter of bacterial response to extracellular polycations, for at least all of the tested polycations. Based on the similarity that we observe between the Min oscillations and previous studies of penetration of these cations into the cell, as well as the cytoplasmic nature of the Min oscillation, we believe that the slowing of the Min oscillation follows polycation penetration to the cell interior.

## Materials and Methods

### Flow cell

**Figure 1:**
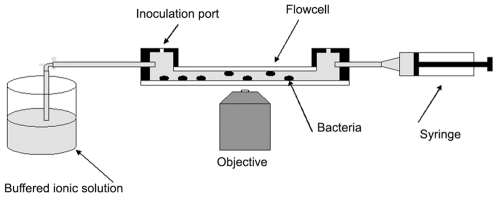
Description of the flow cell. Experimental chamber used for the observation of MinD oscillations in the presence of cations and antimicrobial peptides, as described in the text. Bacteria were observed over a 18 mm×13 mm area on the chamber bottom. The field of illumination and view per image was 0.25 mm^2^, so that a large number of non-overlapping images could be taken.

Experiments were carried out in flow cells with dimensions of 18×13×0.8 mm, as illustrated in Fig. 1. The bottoms of the flow cells consisted of microscope cover slips that were supported by thin metal plates with openings for viewing and imaging of the bacteria. The flow cells were inoculated with bacteria through a small rubber plug. Prior to inoculation the cells were filled with control solution. This was either un-buffered 5 mM NaCl solution or 10 mM HEPES buffer. After inoculation, flow cells were flushed with buffer and remaining bacteria were allowed to settle in the flow cell for at least an hour in order to enhance the number of bacteria attached to the cell bottom. Following bacterial attachment, cationic solution was drawn through the cell with a syringe. Preliminary tests with dyed water showed that all visual traces of the dye disappeared after pulling 20 ml of fluid through the flow cell. All experiments were therefore carried out with that quantity of ionic solution. Fluid exchange flexed the thin bottoms of the chambers, temporarily moving attached bacteria out of focus. Depending on the size of the opening in the cover slip support plate, and hence the degree of cover slip flexing, imaging was delayed for 2–10 minutes after fluid exchange to allow the chamber bottom to flatten. This delay also ensured that ion diffusion was given more than sufficient time to homogenize the extracellular environment in the boundary layers (of thickness of a few microns) at the flow cell walls. A small thermocouple near the flow cell monitored the ambient temperature, between 24 and 26°C, during experiments.

### Strains and growth conditions

Strains of GFP-MinD producing rod-shaped and filamentous *E. coli*, PB103(λDR122) (P*_lac_*::*gfp-minDE*) and PB114(λDR122)/pJE80 (Δ*minCDE* P*_lac_*::*gfp-minDE* P*_ara_*::*sfiA*), respectively, were provided by Piet de Boer [3] and the standard protocols were used for these strains. Unless noted, all cells were grown overnight at 37°C in LB medium. For strain PB103 samples were grown for approximately 14 h at 37°C with added 25 µg/ml of chloramphenicol. A few drops of this suspension were added to a test tube of new medium along with 50 µM isopropyl-β-d-thiogalactopyranoside (IPTG) and, for the filamentous strain PB114, 0.1% of arabinose promoter and cultures were then grown for an additional four hours at 37°C. Approximately 0.5 ml of the new suspension was then injected into a flow cell loaded with control solution and the flow cell was subsequently flushed as described above. Inoculant was also prepared by centrifugation of the new suspension which was then followed by replacement of the supernatant with fresh control solution. Calcium and magnesium test solutions were obtained by dissolving appropriate amounts of CaCl_2_ or MgCl_2_ in de-ionized water. Solutions of protamine or gentamicin were obtained by dissolving these cations in de-ionized water and then adding appropriate amounts of these solutions to the 10 mM buffer or to buffers that contained the desired amounts of Ca^++^ or Mg^++^. We also observed period lengthening effects from divalent ions and polycationic antimicrobial agents when bacteria were suspended in the minimal salt medium M9. However, to avoid possible interference from ions in M9 medium, flow cell studies were performed on bacteria under starvation conditions and in an environment that contained only control solution and the desired cations.

### Fluorescence measurement

Cells were viewed on a Leica DMIRE2 inverted optical microscope outfitted with a Hamamatsu ORCA 285 digital charge-coupled-device camera and a 63× objective (numerical aperture 0.9). A mercury arc lamp provided fluorescence excitation light via a 450- to 490-nm excitation filter, and a 500- to 550-nm barrier filter allowed green fluorescent protein fluorescence imaging. To automatically record several cycles of the MinD oscillations, shutters were placed in the path of the condenser light and the mercury excitation light. The shutters (MAC 5000) were controlled from an Apple iMac 1.8-GHz computer using Open Lab 4 software. Fluorescence images were captured at 1 s or longer intervals depending on the length of the oscillation period to be recorded. Photobleaching was minimized by keeping exposure times short, generally between 30 and 200 ms. Measurement of the oscillation period for a time-lapse series of fluorescence images was done for bacteria that were localized at the bottom of the sample chamber. The oscillation period was determined from a measurement of the average fluorescence intensity in a circular region near one pole. For completely immobilized bacteria either pole was chosen. For partially immobilized bacteria, where the bacterium rotated about one pole, the stationary pole was chosen. The diameter of this circular region was chosen to be approximately the bacterial diameter so that most of the polar intensity could be captured. Fluorescence images were analyzed with the help of Openlab 4 software. In an image sequence, Openlab 4 software automatically places the circular region of interest over the selected pole of a selected bacterium and thus automatically generates the data set for the polar intensity as function of time. Period analysis was performed independently for each bacterium with a least square fit of the intensity function 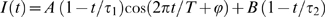 to the intensity data set determined by Openlab 4. Here A, T, ϕ, B, 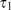, and 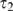 are fit parameters and t is time. In the intensity function the sinusoidal term accounts for the oscillations, the B term for background, and the terms linear in time for moderate photobleaching in the MinD and in the background. The period, T, of this best fit curve was taken as the oscillation period. Except for the longest periods, each time-lapse fluorescence series extended for at least two full oscillation periods.

A typical fluorescence image contained from 20 to 30 bacteria. At low cation concentrations as few as 20% of these were immobilized and the rest, although at the chamber bottom, were sufficiently mobile that the time dependence of their polar intensity could not be recorded. In that case several different positions on the chamber bottom were imaged and an average period was calculated using only immobilized bacteria. In the presence of cations the number of immobilized bacteria increased and a much larger fraction of the visible bacteria could be measured and their periods averaged. In general, periods at a particular ion concentration were calculated from an average over 6 to 20 individual bacteria. Some measurements in the pH range of 5.4 to 5.8 (unbuffered) were made in the presence of 5 mM NaCl while most measurements in this pH range and at the physiological pH of 7.0 were performed in 10 mM HEPES buffer without NaCl. Fluid exchange in the flow cell started with the bacteria suspended in pure control solutions and then proceeded to progressively higher cation concentrations. After the highest concentration the bacteria were returned to the pure control. The process of fluid exchange at low or zero cation concentration often dislodged bacteria from the surface. For elevated cation concentrations, however, a sufficient number of bacteria remained attached during fluid exchange that the period of individual bacteria could be followed as the cation concentration was varied. The oscillation periods were therefore determined by either following individual bacteria or by calculating average periods for a population of stationary bacteria. In all cases, the error bars shown are standard errors.

## Results

### Period determination

**Figure 2:**
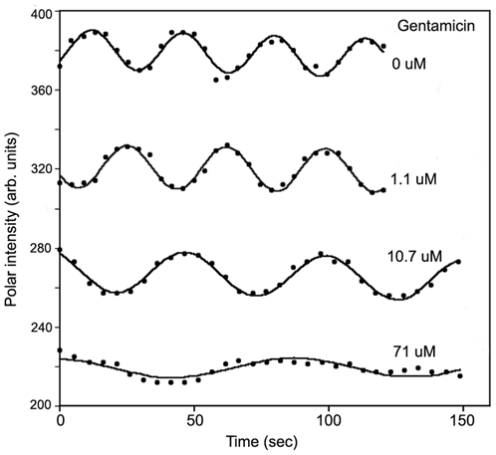
Effect of cations on the MinD oscillation periods of one single *E. coli* PB103 cell. Dots represent experimental polar intensity while solid lines are least square fits to the intensity data. Sequential gentamicin concentrations of 1.1 µM, 11 µM and 71 µM changed the period of this cell from 32 s (no gentamicin) to 37 s, 52.5 s, and 89 s respectively. At high cation concentrations the amplitude of the oscillations decreased and so periods above 200 s could not be measured reliably. The curves for gentamicin concentrations below 71 µM have been offset upwards for clarity: all curves have approximately the same background (non-oscillating) intensity.

Figure 2 shows a typical example of period lengthening when a single bacterium was sequentially exposed to gentamicin for concentrations ranging from 0 µM to 71 µM at pH 7.0. The solid lines are least square fits of I(t) to the polar intensity data. Even for the longest periods, where the quality of the fit was the worst, the fitted period did not depend significantly on the details of the fit function. At low cation concentration several oscillation periods could be recorded. At high concentrations the amplitude of the intensity variations typically decreased–indicating that fewer MinD proteins participated in the oscillations. To avoid excessive photobleaching only one to two oscillation periods were generally recorded at higher concentrations. The error in the period determination depended on the number of periods captured. For 3 or more periods, period errors were less than one second. For periods of more than 100 s with fewer recorded cycles and with generally weaker fluorescence, we estimate period errors of 3 to 4 seconds. Errors in period measurements of a single bacterium, as estimated by variability in times between, e.g., subsequent maxima of the oscillation, were much smaller than the standard errors for groups of bacteria–as estimated by the statistical variation between different bacteria in the same conditions. This indicates significant cell-to-cell variability.

### Phototoxic slowing of MinD oscillations

**Figure 3:**
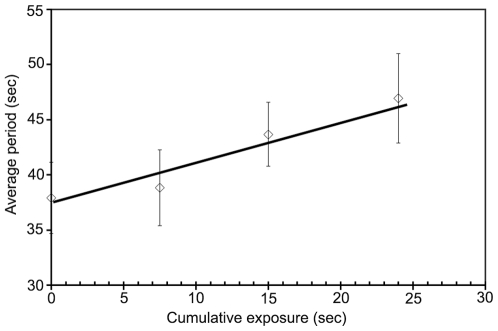
Effect of excitation illumination on MinD periods. MinD oscillation period in 10 mM HEPES buffer at pH 7.0 as function of cumulative exposure time to excitation illumination. The cumulative exposure time represents the sum of the exposure times used for all images taken of a group of bacteria. The time interval between exposures in a sequence of images was 4.5 seconds (to determine the period) and the interval between repeated sequences was 10 minutes (to recover the steady-state response to previous illumination). Repeated exposure of a group of bacteria to the fluorescence excitation light lengthened their average GFP-MinD oscillation period.

**Figure 4:**
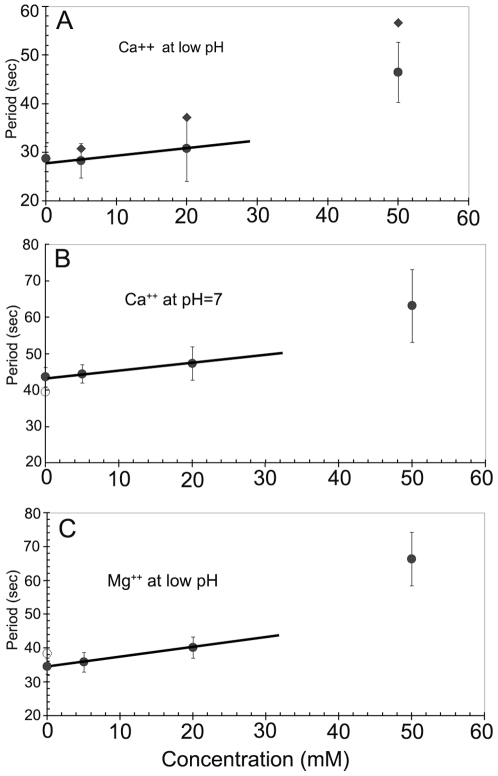
Effect of divalent cations on MinD oscillation periods in *E. coli* strain PB103. Fluorescence images of bacteria were recorded 12–15 minutes after introduction of a new ion concentration into the flow cell. (A) Effect of Ca^++^ ions at an un-buffered pH of 5.5 to 5.8 in the presence of 5 mM NaCl. Raw period data (filled diamonds) have been corrected for cumulative excitation illumination effects (filled circles), as discussed in the text. At 100 mM of Ca^++^ (data point not shown) bacterial fluorescence was uniform over the cell and no oscillating component was observable. (B) Effect of Ca^++^ at a pH of 7.0 in 10 mM HEPES buffer (filled circles). The effects of Ca^++^ cations were reversible, and the original period (open circle) was recovered upon Ca^++^ removal. (C) Effect of Mg^++^ ions on the MinD oscillations at low pH in 10 mM HEPES buffer (filled circles). On return to pure buffer the oscillations returned to their initial value (open circle). There is an approximately linear response of the oscillation period to moderate concentrations of extracellular Ca^++^ or Mg^++^, as indicated by the solid lines.

Small increases in the MinD oscillation period were observed in 10 mM HEPES buffer in the absence of any cations, proportional to the cumulative amount of 450- to 490-nm excitation illumination. When the illumination and viewing region was shifted to unexposed bacteria, shorter periods were again recorded. In Fig. 3 we show the oscillation periods as function of cumulative exposure time for a group of bacteria. To determine the oscillation period the exposure would typically be 6000 ms: 30 exposures with an exposure time of 200 ms each. For cumulative exposures of 25000 ms the period increase is about 10 s. This photon-induced period lengthening was avoided for experiments involving multiple cation concentrations and/or multiple time-points by imaging different groups of bacteria for each period determination. To measure multiple periods in a single bacterium we used short exposures of 50 ms to 100 ms together with the minimum number of images needed for period determination. For data taken before this effect became apparent (see, e.g., Fig. 4A), we corrected for the phototoxic period slowing using the best-fit line in Fig. 3. When compared, these corrected periods agreed with periods taken with changing fields of view.

### Reversible period increase with extracellular Ca^++^ or Mg^++^

Both Ca^++^ and Mg^++^ ions have a significant effect on the oscillation period. The period variation with concentration was measured at both low and physiological pH. Fig. 4A shows the variation of the average period of groups of bacteria (filled circles) at an un-buffered pH∼5.5 up to a maximum Ca^++^ concentration of 50 mM. Error bars indicate the standard error. The period changes at pH = 7.0 in the presence of 10 mM HEPES buffer, shown in Fig. 4B, were similar to those in the un-buffered low pH medium. Experiments with Mg^++^ gave results similar to those for Ca^++^ and an example at low pH is shown in Fig. 4C. For the data in Fig. 4, the divalent ion concentrations were increased from 0 to 50 mM over 45 minutes and MinD oscillation periods showed net increases for both Ca^++^ and Mg^++^ ions. Observation of the oscillations either over longer time periods or upon return to an ion free suspending medium showed that the oscillation period decreased back towards its initial value. This is illustrated by the open circles in Figs. 4B and 4C, which were measured after the 50 mM ionic solutions were replaced by ion free suspending media.

**Figure 5:**
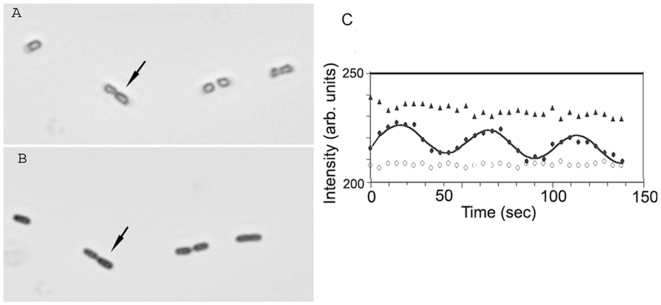
Reversibility of period lengthening for divalent cat ions. (A) Bright field image of *E. coli* bacteria PB103 in 100 mM Ca^++^ ions and 10 mM HEPES buffer. The bacteria assume a more rounded shape and exhibit a bright center region. (B) The same bacteria after the suspending medium is replaced with the 10 mM HEPES control. (C) In 100 mM Ca^++^ the bacterium marked by the arrow in (A) shows a fluorescence that is uniform over its length. Its polar fluorescence is non-oscillatory but decreases slowly with time due to photobleaching (▴). When re-immersed in the control solution, MinD oscillations return for the same bacterium (•, with the solid line as a fit). Open circles indicate fluorescence background levels beside the bacterium (○).

Addition of cations also has more general effects on the bacteria. With 5 mM of Ca^++^ or Mg^++^ ions, bacteria localize to the chamber surface rapidly. At a cation concentration of 20 mM most cell movement has ceased. At concentrations of about 50 mM bright field images of bacteria of strain PB103 suggest that bacteria assume a more rounded shape. At even higher concentrations the bacteria develop a translucent center in bright field images and fluorescence images show that GFP-MinD is either uniformly distributed within cells or is stationary near one pole. MinD no longer appears to oscillate at these very high cation concentrations. Fig. 5A shows a bright field image of such bacteria while suspended in 100 mM Ca^++^. The corresponding polar intensity variation of the bacterium marked by an arrow in Fig. 5A is shown by triangles in Fig. 5C. No MinD oscillation is discernible. When the same bacteria were returned to the ion free control solution, Fig. 5B shows that bacteria recovered their rod shape and the filled circles in Fig. 5C shows that the Min oscillations returned with a period close to the ion free value. The open circles in Fig. 5C represent the background intensity variation for a region next to the bacterium. Addition of high concentrations of Ca^++^ (or Mg^++^) stops the Min oscillations (for at least the one hour observation interval), while subsequent ion removal restarts the oscillation.

**Figure 6:**
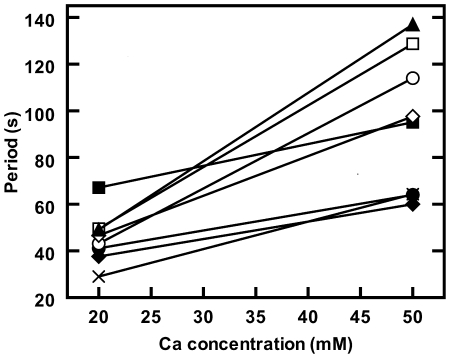
Oscillations as function of Ca^++^ concentration for individual *E. coli* PB103 cells. Period change for eight bacteria when the Ca^++^ concentration was changed from 20 mM to 50 mM at a pH of 7.0. The solid lines are an aid to the eye for each bacterium.

We also followed the oscillation periods of individual bacteria that remained attached to the substrate during all fluid exchange operations. An example of period response for individual bacteria when Ca^++^ concentration was changed from 20 mM to 50 mM is shown in Fig. 6. Although every bacterium in Fig. 6 shows a period increase, the response to Ca^++^ addition shows significant cell-to-cell variability. The larger the extracellular cation concentration the wider the spread of oscillation periods between cells. In contrast to the effects of divalent cations, the addition of monovalent ions such as Na^+^ at up to 5 mM extracellular concentration or measurements in an environment with a high concentration of monovalent salts, such as minimum medium M9 (with over 100 mM monovalent salts), had no significant effect on the Min oscillations.

### Time-dependent response to Ca^++^ or Mg^++^

**Figure 7:**
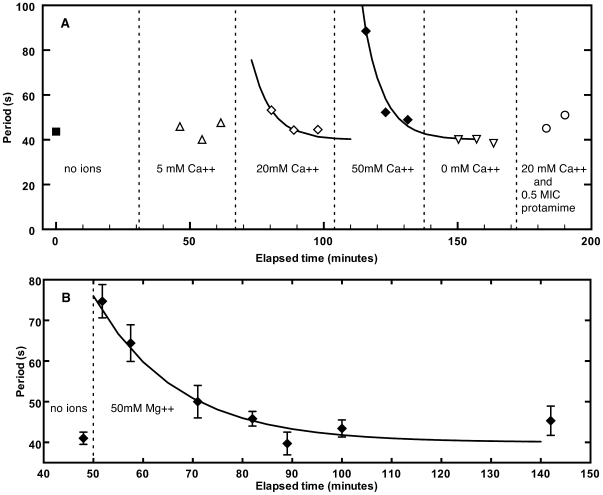
Time response of oscillation periods to changes in Ca^++^ concentrations. (A) Time response of the oscillation periods to Ca^++^ ion concentration changes in 10 mM HEPES buffer at pH = 7.0. The intervals when the ion concentration was constant are indicated. At the beginning of such an interval the ion concentration was increased to the stated value and the first fluorescence measurements were made at this new concentration after a 10 minute delay. Each data point represents the average period of all bacteria in the field of view whose period could be determined. Different data points represent different areas of the sample chamber and hence different groups of bacteria. The two solid lines represent fits of exponential decays to the 20 mM and 50 mM data points. The period decay time for both curves is 7.5 min. The last two points marked by open circles show the periods of two bacterial groups after the chamber was refilled with 20 mM Ca^++^ ions and 0.5 MIC protamine. Effective screening of protamine by Ca^++^ is evident. (B) Example of period changes when *E. coli* bacteria were exposed to 50 mM Mg^++^ in 10 mM HEPES buffer at pH = 5.5. Different data points represent different bacterial groups. Mg^++^ ions were added at the time of the arrow and an improvement of the sample chamber allowed period measurements before the usual 10 minute delay. Solid curve is a fit of an exponential function with a time constant of 16.6 min.

The response of the periods to changes in the ion concentration levels in the suspending medium was time dependent. Fig. 7A shows the time dependence of oscillation periods when groups of bacteria were exposed to different Ca^++^ concentrations at pH = 7.0. The Ca^++^ concentration was increased in three steps (5, 20 and 50 mM) and then returned to zero by filling the chamber with 10 mM HEPES buffer. The time intervals over which the extracellular Ca^++^ concentrations were constant are indicated in the figure. The addition of Ca^++^ produced a rapid period increase that was followed by a decay of the period to values found for the control solutions. Approximate fits of the 20 and 50 mM periods with an exponential function with a decay period of 7.5 minutes are shown as solid lines. For comparison Fig. 7B shows an example of the response of the oscillation period to addition of 50 mM Mg^++^ at a buffered pH of 5.5. As for Ca^++^, the periods for Mg^++^ also increase initially and then decrease again towards values obtained for ion-free control solutions. The decay time in Fig. 7B for the Mg^++^ periods was 16.6 minutes.

For both Ca^++^ and Mg^++^ the onset of period decay was occasionally delayed by as much as 30 min, though reliable statistics were not obtained on this phenomenon.

### Effect of gentamicin

**Figure 8:**
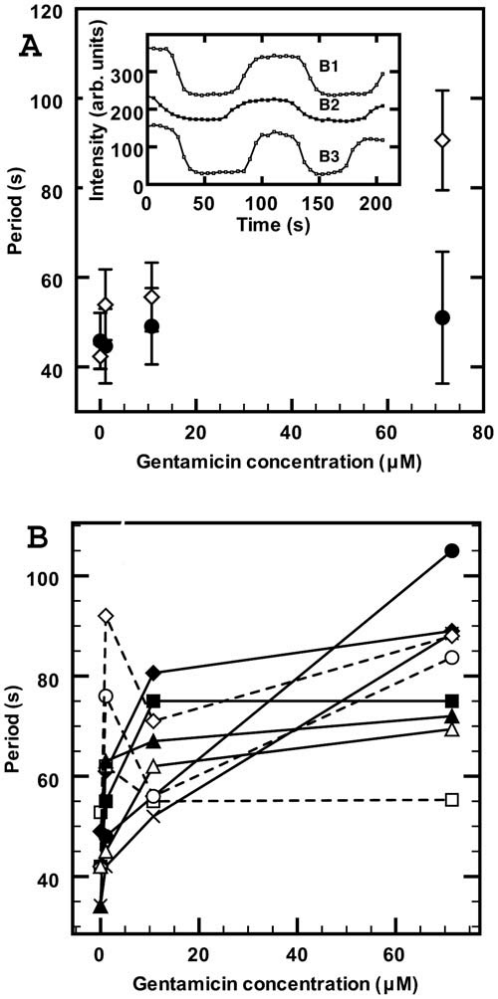
Variation of MinD oscillation period with gentamicin. (A) In 10 mM HEPES buffer at pH of 7.0, the period increases with gentamicin concentration (open diamonds). As shown in the inset, for three bacteria labeled B1, B2 and B3, for gentamicin concentrations of 70 µM or higher the wave form of polar intensity oscillations often became more like square-waves, where the intensity at the poles remained stationary for longer time periods. In the presence of 10 mM HEPES buffer and 20 mM Ca^++^ the oscillation period increase (filled circles) is that expected for 20 mM Ca^++^ alone and periods were independent of the gentamicin concentration within our measurement error. (B) Oscillation periods as function of gentamicin concentration (no added Ca^++^) for nine bacteria at a pH of 7.0. The period increases rapidly at low concentrations for all bacteria. Changes in the period on further increase in the gentamicin concentration varied widely in both sign and magnitude. The general trend was for the period to increase with increasing concentration, but some bacteria exhibited occasional decreases (dotted lines). Lines are an aid to the eye for each bacterium.

The aminoglycoside antibiotic gentamicin also produced considerable period lengthening. Addition of gentamicin at about 5 µg/ml (10.7 µM) immediately reduced the mobility of bacteria in the flow cell and increased their average oscillation period. Period results for gentamicin in 10 mM HEPES buffer at pH = 7.0 are shown in Fig. 8A (open diamonds). The periods, averaged over groups of bacteria, tended to increase rapidly initially with low gentamicin concentration and then rose more slowly as the concentration was further increased to 71 µM. At this concentration the bacteria started to exhibit more rounded shapes but these shape changes were not as pronounced as for the Ca^++^ effects at 100 mM shown in Fig. 5A. We also observed a striking non-sinusoidal Min oscillation at 71 µM of gentamicin, as shown in the inset in Fig. 8A, for three individual bacteria. The oscillation traces appear more like square waves. Similar non-sinusoidal oscillations were occasionally observed for high Ca^++^ levels. Unlike for Ca^++^ and Mg^++^, the period changes for gentamicin were not reversible. Return to control solution did not shorten periods even after four hours. Fig. 8B shows the variation of oscillation periods with gentamicin concentration for 9 individual bacteria that remained localized during all ion exchanges. Although all bacteria show an initial period increase at low concentration, subsequent changes were bacterium dependent with the occasional bacterium at high gentamicin concentration even having a faster period (open squares and open diamonds).

We investigated the effects of Ca^++^ when bacteria are exposed to gentamicin. These results are also shown in Fig. 8A (solid circles). When different gentamicin concentrations in 10 mM HEPES buffer are present together with 20 mM Ca^++^ then only the same small period increase, attributable to the Ca^++^, is observed for any gentamicin concentration. The presence of Ca^++^ ions appears to screen Min oscillations from the effects of this antimicrobial agent (on average). This screening effect was not seen if the Ca^++^ was added after the gentamicin.

### Effect of protamine

**Figure 9:**
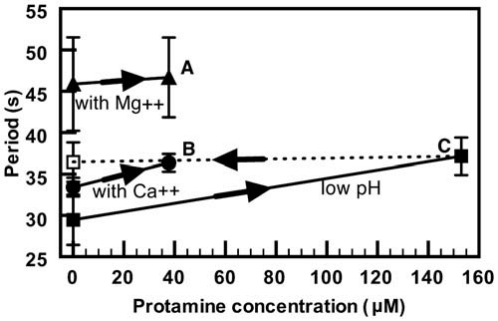
Effect of protamine on MinD oscillations in the presence of divalent cat ions. In the presence of Ca^++^, Mg^++^ or at low pH, the addition of protamine produced only small period changes. (A) 10 mM HEPES at pH 7.0 with 20 mM Mg^++^ and 37.5 µM protamine (▴); no significant period change is observed compared to the protamine free 20 mM Mg^++^ buffer. (B) 10 mM HEPES buffer at pH 7.0 with 20 mM Ca^++^ and 37.5 µM of protamine (•). Only a small period increase is observed compared to the protamine free 20 mM Ca^++^ buffer suspension. (C) Un-buffered control solution at pH near 5.6 with 5 mM NaCl. Increase of protamine concentration from 0 to 153 µM resulted in only a small increase in the oscillation period (▪). The periods in (C), did not change on return to the protamine free control solutions (------). In contrast, the same protamine concentration in the absence of Ca^++^ or Mg^++^ and at a pH of 7 resulted in immediate elimination of MinD oscillations (not shown). Standard errors for all data points are as indicated at “C”.

Unlike Ca^++^ and Mg^++^, the effect of protamine on the oscillation periods was strongly pH dependent (gentamicin was not examined in this respect). At an unbuffered pH around 5.6, addition of protamine at 630 µg/ml increased the periods by only 7 s (Fig. 9, solid squares). No further increase beyond this value was observed, even after one hour. However, as indicated by the dotted line in Fig. 9, 20 minutes after return to the control medium the periods had not recovered. The same small period increase was observed when protamine at 310 µg/ml was added to HEPES buffered medium at pH = 6.0. At a buffered pH of 6.7 or higher, however, addition of 155 µg/ml (37.5 µM) protamine stopped all oscillations in a time shorter than our 10 minute measurement delay. Under these conditions, the MinD fluorescence was either delocalized or frozen at one pole. Return to the protamine free control medium after exposure to 37 µM protamine did not recover the MinD oscillations.

Ca^++^ or Mg^++^ ions significantly screened the action of protamine. Fig. 9 shows the period increase when bacteria were suspended in 10 mM HEPES buffer at pH = 7.0 in the presence of 20 mM Ca^++^ and 37.5 µM of protamine (filled circles). The bacteria were first exposed to the 20 mM divalent cationic solutions. The solutions were then replaced by 20 mM ionic solutions that also contained the appropriate amount of protamine. In the presence of 20 mM Ca^++^ ions the protamine only induced a small period increase, and did not terminate the oscillations. Similar screening effects of protamine action were observed for 20 mM Mg^++^ ions as shown in Fig. 9 (triangles). Single-cell response to protamine was not investigated.

### Cation response of filamentous strain PB114

We also examined the filamentous strain PB114 for period response to protamine exposure. The results for this strain were similar to those found for the rod-shaped strain PB103. In the absence of Ca^++^ at a buffered pH above 6.7, oscillations were rapidly halted by 310 µg/ml of protamine though fluorescence patterns of some bacteria appeared frozen in time–retaining the characteristic spatial modulation with a wavelength of about 8 microns [3]. In other cells the fluorescence was more uniformly distributed. In contrast to strain PB103 where cell deformation towards a spherical shape was induced by protamine addition, the filamentous bacteria maintained their shape as resolved under bright field.

## Discussion

### Slowing of Min oscillation by Ca^++^ or Mg^++^

The introduction of extracellular Ca^++^ or Mg^++^ significantly slows Min oscillations in *E. coli* (Fig. 4). This slowing was accompanied by an increased cell-to-cell variability of the oscillation period (Fig. 6). After the initial increase, the period relaxes back towards ion free values with decay times of approximately 8 min for Ca^++^ and 17 min for Mg^++^ (Fig. 7), though occasionally the onset of this decay is significantly delayed. At extracellular concentrations of 100 mM or more, oscillations were frozen until concentrations were returned to lower values.

The time-dependent response of the periods when cells were exposed to extracellular Ca^++^ is similar to the time dependence of intracellular Ca^++^ levels as determined in experiments using aequorin. In those studies, introduction of 1–10 mM of extracellular Ca^++^ resulted in immediate increases of [Ca^++^]_i_ followed by rapid recovery towards normal cytoplasmic levels, as well as a much slower oscillatory response of [Ca^++^]_i_ [14], [15]. All cells had significant response to extracellular cations. However, we did find considerable single-cell variability with some (but only some) of the cells observed even exhibited period decreases upon increasing cation concentrations (Fig. 8B) or exhibited significant delays before the onset of the decay of changed oscillation periods. Single-cell variation in the *timing* of actively regulated response to cations could explain the cytosolic free calcium oscillations observed in *E. coli* [14] together with the increased variability observed with increased cation concentration (Fig. 6). In light of our results, detailed investigations of how the initial cation response and subsequent relaxation depends, for single cells, on parameters such as ionicity, growth medium or bacterial growth phase are needed and should now be possible.

### Possible mechanisms of action by polycations on Min oscillations

Any hypothesized mechanisms for direct effects of polycations on the Min oscillation should be consistent with the observed similarity of response to a wide variety of polycations. The dynamic molecular processes that generate MinD oscillations have been described in detail in the literature [1] and fall into four spatially-coordinated steps in normal rod-shaped cells: i) ATP-bound MinD (cooperatively) associates with the cytoplasmic membrane to “cap” one pole of the cell; ii) it then recruits cytoplasmic MinE (and MinC) to the membrane; iii) the bound MinE stimulates the MinD ATPase and MinD-ADP (and associated MinE and MinC) are released to the cytoplasm; iv) subsequent nucleotide exchange allows this cycle to periodically repeat at alternate poles.

Min oscillations appear to be rate limited by the disassembly of the MinD polar caps, i.e. by the MinE-stimulated release of membrane-associated MinD. We infer this from the observations that new MinD polar caps form as the previous one is still disassembling [10], that new MinE rings form without appreciable lag after the previous one disassembles [27], [28], and that the dynamics of the MinD polar cap is symmetric in time between assembly and disassembly (see, e.g., Fig. 2). If so, then slower periods indicate that cations *decrease* the MinE-stimulated MinD ATPase activity—since this controls MinD polar cap disassembly. This might occur by cation-dependent changes of the stimulated ATPase activity of bound MinE, similar to its postulated strong temperature dependence [5], or by reduced affinity of MinE to MinD filaments. However it could also be due to nonspecific cationic bundling and subsequent stabilization of MinD polymers (see, e.g., [29]), or to nonspecific aggregation (see, e.g., [30]) of MinD and/or MinE leading to reduced ratios of MinE to MinD participating in the subcellular Min oscillation. It seems reasonable to assume that such cation effects on Min oscillations require the presence of the cations on the cytoplasmic side of the plasma membrane or, in other words, cation penetration to the cytoplasm. Indeed, cations cannot directly influence Min oscillations from outside the cell or even from outside the inner bacterial membrane, due to strong electrostatic screening.

The observed “squaring” or freezing of Min oscillations at high cation concentrations could be qualitatively explained by any of these direct cytoplasmic mechanisms. However, our observation that a decreasing amount of MinD participates in oscillations as cationic concentrations increase seems to support the non-specific aggregation hypothesis. There are doubtless other plausible direct or indirect mechanisms. Studies of GFP-MinE are needed to see whether the MinE ring visibly weakens as the Min oscillation slows, as would be expected for the non-specific aggregation mechanism in leading to slower oscillations.

### Transport of antimicrobial cations

Protamine, a cationic antimicrobial peptide, and gentamicin, an aminoglycoside, led to halted (with neutral pH and without Ca^++^ or Mg^++^) or lengthened (Figs. 2, 8, and 9) Min oscillations. While these effects were irreversible over observation times of several hours, they were not accompanied by cell lysis. Furthermore, the effects were significantly reduced in the presence of tens of mM Ca^++^ or Mg^++^. The effects of cationic antimicrobial agents on MinD oscillations parallel the effects of protamine on the growth of bacteria [26] in dosage, in pH dependence, and in the inhibitory effects of Ca^++^ and Mg^++^.

Our observed reversion of the MinD oscillation period (homeostasis, with a timescale comparable to the measured cytoplasmic homeostasis of [Ca^++^]_i_ [15]), despite the consistently high periplasmic Ca^++^ levels associated with extracellular Ca^++^ [31], indicates that cations outside the cytoplasm do not indirectly affect the Min oscillation. Our observations of irreversibility of the protamine and gentamicin effects on the MinD oscillation, even after the extracellular medium is replaced by pure buffer, also support this conclusion. To influence MinD, which associates with the cytoplasmic side of the inner bacterial membrane, we believe that cations traverse the outer membrane and periplasmic space and penetrate the inner membrane into the cell interior. Indeed, extracellular Ca^++^ [14], [15], [31], [32] and gentamicin [33] have previously been directly shown to penetrate into the cytoplasm, and Mg^++^ is associated with active (uptake) transporters [16], [17]. Cytoplasmic penetration is consistent with the observation that Ca^++^ [12], Mg^++^ [17], and gentamicin [34] are all associated with known cytoplasmic efflux systems. Indirect evidence also points to efflux systems that act on protamine such as the CmeABC system of *Campylobacter jejuni* [35].

Divalent cations Ca^++^ and Mg^++^ can also have a strong influence on the action of antimicrobial peptides (see, e.g., [23]) and of gentamicin [36], [37]. For example, more than 10 mM Ca^++^ significantly increases the MIC for protamine [23]. Simulations showed that the highly charged protamine could not cross the bacterial outer membrane when significant concentrations of Mg^++^ or Ca^++^ ions were also present [23], and this has been confirmed *in vitro* with Ca^++^ [38]. This exclusion of protamine from the outer bacterial surface by Ca^++^ or Mg^++^ would explain the absence of significant MinD period lengthening by protamine in the presence of 20 mM Ca^++^ or Mg^++^. We hypothesize that this mechanism also applies to gentamicin.

Our results indicate that both protamine and gentamicin affect the Min oscillation without lysis. The absence of lysis in *E. coli* cells upon moderate protamine addition was already determined previously [26] and is confirmed by our observation that the average GFP fluorescence intensity remained unchanged upon protamine addition, unlike the rapid decrease of GFP fluorescence observed after rapid mechanical rupture using micromanipulators (data not shown).

The metabolic state of bacterial cells can influence the activity of antimicrobial peptides. For example, protamine susceptibility in *E. coli* depends on the pmf of the cytoplasmic membrane: a low membrane potential, as observed for high acidity environments, leads to decreased protamine sensitivity of cells [39]. The pH dependence of the protamine sensitivity that is observed in cell growth experiments is similar to the pH dependence of our Min oscillation periods. However, the membrane potential is unlikely to have a direct effect on oscillation periods since we found that, in the absence of polycations, periods were independent of pH. Rather we believe pH dependence controls the penetration of polycations into the cell and that those cations then affect the Min oscillations. Indeed, uptake of aminoglycosides by *E. coli* membrane vesicles was previously shown to be controlled by the pH dependent membrane potential Δψ [40], [41]. Uptake of the aminoglycoside tobramycin was furthermore shown [40] to be consistent with presence of voltage-gated channels on the cytoplasmic membrane, though whether this mechanism of entry is used by the cations in our study has not been determined.

While we believe that the best explanation of our results is that all of the tested polycations have penetrated to the cytoplasm, it is certainly true that the changing period of the Min oscillation is a fast single-cell cytoplasmic marker of the action of the tested polycations. If the Min oscillation generically responds to polycations, then it may prove useful in antimicrobial drug development as a fast reporter of penetration and/or effect.

### Min oscillations as a reporter of polycations

While we were only able to obtain quantitative period measurements 2–10 minutes after fluid transfer with our home-built imaging chambers, better chamber designs and higher temperatures [5] should shorten the blackout period: qualitatively the response of the Min oscillation is faster than one Min oscillation. Min oscillations thus appear to be a fast indicator for sublethal cation exposure. Min oscillation can be observed in single bacterial cells and the oscillatory signal is easily distinguished from background fluorescence (see, e.g., Fig. 2 and Fig. 5C). As a result, Min oscillations offer a single-cell reporter of bacterial response due to extracellular polycations, which we think is due to the cations being exposed to the cytoplasm. This single-cell response is in contrast with, e.g., the photoprotein aequorin, which does not provide single-cell sensitivity. However, the Min response is not calibrated and its mechanism is (as yet) undetermined. Indeed, it is not yet clear whether the significant cell-to-cell variability in the response of the Min oscillations that we observed is due to variable cation penetration and/or effect, due to variable Min protein expression, or due to a combination of the two.

Min oscillations exhibit similar sensitivity to Ca^++^ and Mg^++^, despite the thousandfold difference in their typical cytoplasmic concentrations. This may be because the Min oscillation is endogenous to *E. coli*, so that the moderate scales of responses of the Min system are similar for typical extracellular challenges. This may also underlay the conveniently large dynamic range of the Min sensitivity for all of the cations examined, which extends up to concentrations of cations that start to affect growth systemically.

The phototoxic period lengthening observed when single cells were repeatedly imaged is inconvenient. While individual exposures shorter than 50 ms will minimize phototoxicity, it might be avoided altogether with the use of a non-phototoxic buffer (unlike HEPES [42]). Alternatively, if the phototoxicity arises from photobleaching of the GFP fused to MinD, then the use of fluorescent MinC fusions should reduce the phototoxicity. (MinC follows the Min oscillation [2], [43] but does not influence it [3].) Preliminary indications (data not shown) are that observed phototoxic slowing were due to the HEPES buffer [42].

### Experimental control of Min oscillations

We have observed several distinct effects on Min oscillations due to extracellular polycations: the slowing of the oscillation period and the decreasing amplitude of the MinD oscillation with increased concentration, the distortion of the oscillation with intermediate concentrations, and the freezing of the oscillation with very high concentrations. These effects were seen with Mg^++^, Ca^++^, protamine, and gentamicin—all polycationic by otherwise quite different in size and shape. The observed reversible freezing of the Min oscillations has not been previously observed experimentally, despite being a common prediction of quantitative models. Refined studies of this reversible freezing should enable watching the initial growth of the Min oscillation instability.

The control of Min oscillations by the cations Ca^++^, Mg^++^, gentamicin, and protamine, extends previous studies that showed physiological effects of, e.g., Ca^++^ in protein expression [44], Ca^++^ and Mg^++^ in cell adhesion [45], and antimicrobial peptides in various physiological processes [46], [47]. Manipulation of extracellular cations, cell geometry (of filamentous cells using micropicks, data not shown), and temperature [5] are now in the “toolbox” for perturbing Min oscillations *in vivo*. We hope that by combining and refining these approaches, and by using them to test and develop computational models, we will obtain more insight into the remarkable subcellular Min oscillation.

### Summary

This paper reported effects of extracellular divalent cations, cationic antimicrobial peptides, and aminoglycosides on subcellular oscillations of MinD-GFP within *E. coli*. The average Min oscillation period increased with increasing concentration of Ca^++^, Mg^++^, protamine, or gentamicin. At high concentrations oscillations ceased. The period lengthening or freezing of the oscillations for the divalent cations was reversible, and at lower concentrations echoed the previously observed homeostasis of intracellular Ca^++^ in the face of constant extracellular concentrations. Protamine and gentamicin produced non-reversible period increases. Both protamine and gentamicin in the bacterial cytoplasm affect Min oscillations without either lysis (as compared to mechanical cell rupture) or cell death (as witnessed by the ongoing Min oscillation at lower cation concentrations). Moderate amounts of divalent cations in the extracellular medium strongly reduced the effects of both protamine and gentamicin on the oscillation period, apparently by preventing them from entering the cell. The photon yield from a single bacterium is sufficient that oscillation periods can be measured on individual bacteria over a range of ion concentrations. We believe Min oscillations are responding to cytoplasmic cations, so that Min oscillations might therefore serve as an effective single-cell reporter of intracellular polycations. However, further work needs to be done to validate this hypothesis through confirming the mechanism(s) of action. Further study of the effects of extracellular cations on Min oscillations–particularly the transition into and out of a non-oscillating state–should also lead to a better understanding of the mechanisms that drive and control these oscillations.
